# Hypoketotic hypoglycemia in citrin deficiency: a case report

**DOI:** 10.1186/s12887-020-02349-6

**Published:** 2020-09-22

**Authors:** Yoichi Wada, Natsuko Arai-Ichinoi, Atsuo Kikuchi, Osamu Sakamoto, Shigeo Kure

**Affiliations:** 1 Department of Pediatrics, Tohoku University School of Medicine, 1-1 Seiryomachi, Aobaku, Sendai, Miyagi 980-8574 Japan; 2 Tohoku Medical Megabank Organization, 2-1, Seiryomachi, Aobaku, Sendai, Miyagi 980-8573 Japan

**Keywords:** Citrin deficiency, Hypoketotic hypoglycemia, Medium-chain triglyceride, Starvation test, *SLC25A13*

## Abstract

**Background:**

Citrin deficiency (CD) is a recessive metabolic disease caused by biallelic pathogenic variants in *SLC25A13*. Although previous studies have reported ketosis in CD, it was observed at the time of euglycemia or mild hypoglycemia. Blood ketone levels concomitant with symptomatic or severe hypoglycemia in CD have not been a topic of focus despite its importance in identifying the etiology of hypoglycemia and assessing the ability of fatty acid utilization. Herein, we describe a patient with CD who had repeated episodes of hypoglycemia with insufficient ketosis.

**Case presentation:**

A 1-year-old boy with repetitive hypoglycemia was referred to us to investigate its etiology. The fasting load for 13 h led to hypoketotic hypoglycemia, indicating the possibility of partial β-oxidation dysfunction. A genetic test led to the diagnosis of CD. The hypoglycemic episodes disappeared after switching to a medium-chain triglyceride-containing formula.

**Conclusions:**

This case report suggests that symptomatic or severe hypoglycemia in patients with CD could be associated with relatively low levels of ketone bodies, implying that β-oxidation in these patients might possibly be partially disrupted. When encountering a patient with hypoglycemia, clinicians should check blood ketone levels and bear in mind the possibility of CD because excessive intravenous administration of glucose can cause decompensated symptoms in patients with CD as opposed to other disorders presenting with hypoketotic hypoglycemia, such as fatty acid oxidation disorders. Further studies in a large-scale cohort are warranted to confirm our speculation.

## Background

Citrin deficiency (CD) is caused by biallelic pathogenic variants in *SLC25A13* (MIM 603859) that encodes citrin, a mitochondrial membrane protein that is mainly expressed in the liver [1, 2]. Citrin protein exchanges aspartic acid with glutamic acid between the mitochondria and cytosol to maintain NADH and NAD^+^ balance [3]. The NADH/NAD^+^ ratios increase in the cytosol in patients with CD [4], resulting in NADH/NAD^+^ imbalance after glycolysis. Individuals with CD have unique preferences for low-carbohydrate and high-fat foods, which are probably associated with energy production balance [5]. CD often markedly causes hypoglycemia during childhood; thus, physicians should consider the possibility of CD in such children. The assessment of ketone body levels during hypoglycemia is crucial for differential diagnosis [6]. A previous study diagnosed this as ketosis based on the results of urine analysis and not those of blood analysis, at the time of the episode of hypoglycemia [7]. Urine ketone tests could overestimate the values owing to abnormal urine concentration and might not explain the appropriate blood ketone status at the time of assessment. However, blood ketone levels concomitant with symptomatic or severe hypoglycemia in patients with CD have not been studied.

Herein, we describe the case of a patient with CD who had repeated episodes of hypoglycemia with insufficient ketosis.

## Case presentation

The patient was born at 37 weeks of gestation without adverse perinatal events and neonatal jaundice. His body weight and height at birth were 2530 g (− 0.39 SD) and 47.0 cm (− 0.63 SD), respectively. Newborn screening tests were negative. At the age of 1 year, he started presenting afebrile convulsions on days when he fell asleep without having milk at night. Laboratory examination indicated hypoglycemia (glucose levels, 0.83 [normal, 3.89–6.11] mM). He was transferred to a previous hospital to investigate the cause of the hypoglycemic episode. His height and body weight were 73.1 cm (− 0.7 SD) and 9.6 kg (+ 0.3 SD), respectively. He had a plump face but no hepatosplenomegaly. After 12 h of fasting, blood samples revealed hypoglycemia without ketosis (glucose, 1.61 mM; insulin, 1.2 pmol/L; total ketone bodies, 477 μM; acetoacetic acid, 87 μM; and 3-hydroxybutylate, 390 μM). Laboratory examination revealed mild liver injury and hypertriglyceridemia (AST, 94 [normal, 23–57] U/L; ALT, 66 [normal, 9–38] U/L; and TG, 8.81 [normal, 0.23–1.69] mM). The blood ACTH, cortisol, IGF-1, thyroid hormones, galactose, and amino acid levels were normal. Urine organic acid and serum acylcarnitine analyses with hypoglycemia showed nonspecific results. Glycogen storage disease (GSD) type Ia was suspected on the basis of these laboratory findings. The patient was given a high-glucose-containing formula for GSD to avoid hypoglycemia; however, he still presented repetitive hypoglycemia when fasting over 12 h. In total, three episodes of hypoglycemia with glucose levels below 2.5 mM were observed. At the age of 1 year and 1 month, he was referred to our institution to investigate the cause of repetitive hypoglycemia despite the consumption of a formula for GSD. Given the possibility of fatty acid oxidation disorders (FAODs) or GSDs, we measured sequential glucose and ketone levels in a fasting state as well as glucose loading (Table 1). A venous cannula was inserted on the last day; all feeding was stopped at 9 PM, and blood glucose and 3-hydroxybutyrate levels were checked every 3 h until 12 h or every 1 h after 12 h using portable blood glucose or ketone meters. After 13 h of fasting, the baby boy showed signs of hypoglycemia without severe ketosis [6, 8]. Shortly thereafter, he orally consumed 20 mL/kg of 10% glucose solution enthusiastically. His blood glucose levels increased, but his blood lactate levels remained unchanged before and after glucose loading. Serum acylcarnitine levels were mildly elevated in multiple acylcarnitines, which is sometimes observed in hypercatabolism. Urine organic acid analysis revealed nonketotic dicarboxylic aciduria. At the first outpatient visit after the test, the baby’s mother mentioned his food preferences: He particularly liked soybeans. This led to the suspicion of CD. Genetic testing revealed compound heterozygous variants of *SLC25A13* (NM_014251: c.[1019_1177del]; [1813C > T]), both of which are reported as prevalent pathogenic variants [9], leading to a diagnosis of CD. The hypoglycemic episodes and mild liver injury disappeared after switching the high-glucose-containing formula for a medium-chain triglyceride (MCT)-containing formula (AST, 32 U/L and ALT, 16 U/L).

**Table 1:** Laboratory values at the pre-starvation and post-starvation tests

Laboratory Measure	Pre-starvation test	Post-starvation hour			Post-intake hour		2
		10	12	13	0.5	1	
Blood glucose, mM		4.17	3.44	1.89	5.67	7.28	7.78
Lactate, mM		5.40^a^		1.98	1.63	1.36	1.41
Pyruvate, mM				0.10	0.10	0.09	0.13
Acetoacetic acid, μM	40			576	784	820	411
β-Hydroxybutyrate, μM	67	300^a^		1616	1551	1582	646
Total ketone bodies, μM	107			2192	2335	2402	1057
Free fatty acid, mM				4.4			2.5

^a^Measured using a portable blood lactate or β-hydroxybutyrate meter

## Discussion and conclusions

Here, we described the clinical history of a baby boy with repetitive episodes of hypoglycemia without adequate ketone production. The fasting tolerance test showed that the patient demonstrated hypoglycemia without appropriate ketone body elevation. A genetic test revealed pathogenic *SLC25A13* gene variants and led to the diagnosis of CD; the use of MCT-containing formula resolved the hypoglycemic episodes.

We collected serial blood samples during a starvation test, allowing us to exactly determine the ketone body dynamics in response to hypoglycemia. Inappropriately low levels of ketones in the presence of severe hypoglycemia may provide a diagnostic clue for CD. Characterizing the metabolic profile using accessible and convenient laboratory tools could help physicians and patients because CD exhibits nonspecific symptoms and has little diagnostic evidence [10]. Moreover, it is important to recognize that a hypoglycemic state in CD is associated with insufficient ketone levels because relatively high ketone levels have been reported in patients with CD. A previous case of CD reported as ketotic hypoglycemia was determined using a urine sample, while the blood ketone levels might have been relatively low for blood glucose (0.78 mM) because 2+ result of qualitative tests for urine ketones would be approximately 400 μM of acetoacetic acid [7]. The blood ketone levels might be relatively low in the hypoglycemic situation. Moreover, another study describing ketotic hypoglycemia revealed a CD case with hypoglycemic convulsion [11], while blood ketone levels (total ketone body level was 1.8 mM) were certainly elevated but relatively low for the predicted compensatory change (expected total ketone body level was > 5 mM) [8]. This result should be considered as insufficient ketosis with hypoglycemia. Further large-scale cohort studies are required to verify our speculation.

The underlying mechanism of inappropriately low ketone levels with hypoglycemia in patients with CD is unknown; however, this hypoketotic state may be associated with the altered redox states of both cytosol and mitochondria in CD. The fasting test showed that the 3-hydroxybutylate to acetoacetic acid ratio was lower than expected, and that the reduction of 3-hydroxybutylate may be a secondary effect due to the elevation in cytosolic NADH/NAD^+^. The patient was first suspected of having GSD Ia and prescribed with a high-glucose formula to prevent hypoglycemic episodes. A high glucose level may increase NADH/NAD^+^ levels in the cytosol and disturb the NADH/NAD^+^ balance via glycolysis, and the shift in NADH/NAD^+^ possibly caused hypoglycemia in this patient [12]. The hypoglycemic episodes disappeared after switching from the high-glucose-containing formula to an MCT-containing formula. This could be due to the improvement of NAD^+^/NADH imbalance by MCT supplementation via an increase in the availability of NAD^+^ [13]. In addition, the NADH/NAD^+^ imbalance, which may be exacerbated by high glucose intake, might be associated with mild liver injury long after the infantile period when patients with CD usually present with cholestasis because NADH/NAD imbalance can lead to oxidative stress and damage in the hepatocyte [14].

The partial impairment of β-oxidation might possibly occur and could contribute to hypoketotic condition via peroxisome proliferator-activated receptor (PPAR) dysregulation; this hypothesis might have explained the phenotypic discrepancy between patients with CD and FAODs if these disorders commonly occurred due to errors in β-oxidation. PPARα, a subtype of PPAR, is enriched in the liver. It maintains lipid homeostasis and positively regulates multiple enzymes of β-oxidation [15]. A previous study reported that *PPARA* mRNA expression was downregulated but *PPARD* was not downregulated in patients CD [16]. In PPARs, the downregulation of PPARα and not PPARδ or PPARγ is associated with liver steatosis in patients with CD [16]; in contrast, PPARα seemed to be upregulated in patients with FAODs, such as in those with trifunctional protein deficiency and very-long-chain acyl-CoA dehydrogenase deficiency [17, 18]. *Ppara* knockout mice seemingly did not show any phenotypes; however, they showed hypoketotic hypoglycemia in a fasting condition and could easily affect patients with fatty liver, which is similar to that in CD [19]. Transgenic mice of *Ppara* and not *Ppard* or *Pparg* showed hypertrophic cardiomyopathy, which can result in FAODs [20, 21]. Thus, PPARα dysregulation could be related to pathogenicity in CD. In our patient, urine organic acid analysis revealed nonketotic dicarboxylic aciduria, which is consistent with the partial inhibition of β-oxidation. Moreover, the extremely high free fatty acid levels might result from the partial blockade of β-oxidation or physical and mental burden of the fasting test. MCT might help the hypoketotic state of the patient with CD to overexpress enzymes in β-oxidation [22, 23]. The difference between CD and FAODs may simply be due to the expressed tissues [1, 24]. Additional studies are warranted to confirm the relationship between PPARα dysregulation and phenotypes of CD.

In summary, CD might possibly cause hypoketotic hypoglycemia due to partial impairment of ketogenesis, and physicians should measure blood ketone levels in cases of unexplained hypoglycemia. It is important to consider the possibility of CD during the differential diagnosis of hypoketotic hypoglycemia because high-glucose intake may cause hyperglycemia and exacerbate the metabolic state rather than prevent hypoglycemia.

## Data Availability

The datasets used and/or analyzed during the current study are available from the corresponding author on reasonable request.

## Abbreviations

CD: Citrin deficiency
FAOD: Fatty acid oxidation disorder
GSD: Glycogen storage disease
MCT: Medium-chain triglyceride
PPAR: Peroxisome proliferator-activated receptor
